# In-the-Moment Profiles of Expectancies, Task Values, and Costs

**DOI:** 10.3389/fpsyg.2019.01662

**Published:** 2019-07-17

**Authors:** Julia Dietrich, Julia Moeller, Jiesi Guo, Jaana Viljaranta, Bärbel Kracke

**Affiliations:** ^1^Institute of Educational Science, University of Jena, Jena, Germany; ^2^Institut für Bildungswissenschaften, Leipzig University, Leipzig, Germany; ^3^Institute for Positive Psychology and Education, Australian Catholic University, Strathfield, NSW, Australia; ^4^Philosophical Faculty, School of Educational Sciences and Psychology, University of Eastern Finland, Joensuu, Finland

**Keywords:** situational motivation, diary study, experience sampling method, intensive data, expectancy-value theory, multilevel latent profile analysis

## Abstract

This study focuses on the situational heterogeneity of motivation by investigating in-the-moment profiles of expectancies, task values, and costs within learning situations during a university lecture. In a sample of 155 undergraduate students followed across one semester we examined the occurrence of six hypothesized profiles, situational profile change, and the associations of situational motivation profiles with students’ dispositional motivation. Results of multilevel latent profile analysis revealed three profiles with symmetric levels of expectancies, values, and costs (reflecting high, medium, and low motivation situations), and one profile reflecting motivating but costly situations. Furthermore, situational profiles were associated with students’ motivational dispositions at beginning and end of the semester, and partly related to changes in these dispositions during the semester.

## Introduction

As school-, college- or university teacher, one often notices fluctuations in attention or engagement across a lesson and across a course. Mostly, one attributes inattention to lacking motivation of some students. However, if the group of students is large, like in a lecture, one cannot be sure whether it is the same individuals in each lesson who are not motivated or whether groups of unmotivated individuals have changed. Information about the variety among students’ motivational states during a lesson and the heterogeneity of their development across a course would be helpful to provide differentiated instruction (Tomlinson et al., 2003) that adapts to the needs of different students (Corno, 2008). This study focuses on such situational heterogeneity in motivational states and investigates its interplay with individual motivational dispositions of students.

We base our study in the theoretical frame of expectancy-value theory because task-related value beliefs (“Why should I learn this?”) and expectancies students hold about their success in a task (“Can I learn this?”) are central antecedents of student engagement and their behavior in class (Eccles and Wigfield, 2002). According to expectancy-value theory, task value includes positive components (intrinsic, attainment, and utility values) and negative ones (costs). The term *task* value indicates that the construct was developed to describe the motivation of a person to engage in a specific *task*. Despite this emphasis on specific tasks and situations, most studies using the term task value assess instead the values attributed to general domains or school subjects, rather than those attributed to specific tasks (e.g., Perez et al., 2014; Dietrich et al., 2015; Viljaranta et al., 2017).

In contrast, this study focuses on the momentary experiences of task values and success expectancies that students attribute to specific learning situations. We distinguish between specific facets of situational values, costs, and success expectancies and examine in-the-moment profiles of these facets during a university lecture. We aimed to find out whether the relations among value, cost, and expectancy facets differed across situations. For example, we expected to find profiles of generally high values and expectancies, profiles of generally low values and expectancies, and discrepant profiles of high values and low success expectancies (i.e., difficult but valued tasks), or the opposite discrepant profile of low values but high expectancies (i.e., easy but unimportant tasks).

While a number of recent studies have tackled the motivational heterogeneity of the students in a classroom (e.g., Bråten and Olaussen, 2005; Conley, 2012; Lazarides et al., 2016; Viljaranta et al., 2016; Dietrich and Lazarides, 2019), due to their design these studies were unable to examine the heterogeneity of motivational states within students. Therefore, examining in-the-moment profiles of values, costs and expectancies is new because due to the lack of situational, task-specific measures, most previous studies examined clusters of individuals instead of clusters of situations. To examine the intra-individual, situational profiles of expectancies, values, and costs in the moments in which they occur, we used multilevel latent profile analysis (MLPA). After identifying the situational profiles, we examined their associations with students’ changes in dispositional measures of expectancies, values, and costs in university students who attended a weekly lecture across one semester.

### Definitions of Task Values, Costs, and Success Expectancies

The expectancy-value model of achievement motivation (Eccles and Wigfield, 2002; Wigfield et al., 2009) proposes that achievement behavior is largely influenced by expectancies of success and subjective task values. Both constructs are subdivided into specific facets. *Task values* include intrinsic value (the enjoyment and interest that a person gains from a task), utility value (the usefulness of a task for the pursuit of other short- and long-term personal goals), and attainment value (the relevance of a task to a person’s sense of self, identity, and core personal values; Eccles and Wigfield, 2002). Many empirical studies found the three facets of intrinsic, attainment, and utility values to be highly correlated, which is why some researchers have collapsed them into an overall value scale (e.g., Eccles et al., 1993; Viljaranta et al., 2009; Gniewosz and Noack, 2012; Perez et al., 2014). A fourth task value facet, costs, is defined as the negative aspects that result from engaging in a task (Eccles and Wigfield, 2002). Costs include effort costs (i.e., the effort and hard work required by a task), opportunity costs (i.e., opportunities that are lost because of the engagement in the task), and emotional costs (e.g., feeling worried, anxious, and being stressed; Perez et al., 2014). *Expectancy beliefs* include a person’s task-specific success expectations (evaluation of one’s capacity to succeed in the task), and a person’s broader perceptions of the own competence in a given domain (i.e., self-concept of ability; see Eccles and Wigfield, 2002).

Recent evidence suggests that it is insightful to distinguish between dispositional and malleable, situational components of expectancies and values (e.g., Vancouver and Kendall, 2006; Tsai et al., 2008; Malmberg et al., 2013; Martin et al., 2015; Dietrich et al., 2017). We therefore developed *in situ* measures for expectancies (success expectations and perceived competence), task values (intrinsic, attainment, utility value), and costs (effort cost, opportunity cost, and emotional cost) and applied them in this study. In line with recent studies, we separated costs from the other task value facets (Perez et al., 2014; Flake et al., 2015).

### Profiles of Motivational Experiences

A large amount of studies has investigated the linear relationship between expectancies and values, showing that both are distinct but positively correlated constructs (Denissen et al., 2007; Pinxten et al., 2014; Kosovich et al., 2017), and that this relationship becomes stronger with increasing student age (Wigfield et al., 2009). However, some studies found weak associations between expectancy and value measures in college students, the age group of the present study (e.g., Finney and Schraw, 2003; Hendy et al., 2014).

This variation in findings could be reconciled by the idea that different subgroups might be hidden behind an overall correlation. Several studies suggest that the association between expectancies, task values, and costs, varies between individuals: On the one hand, findings indicate that many learners show profiles of symmetric *high*, *medium*, and *low* motivation, where, for instance, high expectancies go along with high values (Bråten and Olaussen, 2005; Conley, 2012; Lazarides et al., 2016, 2019; Viljaranta et al., 2016, 2017). On the other hand, Pekrun and colleagues’ control-value theory of achievement emotions (Pekrun, 2006) posits that discordant combinations of value and expectancy are possible (e.g., high value combined with moderate or low expectancy is assumed to lead to anxiety and hopelessness, respectively). In line with this, empirical studies revealed that some students do experience discrepancies of either *high value and low expectancy* beliefs (a subject is relevant but difficult), or vice versa (*low value and high expectancy*, i.e., a subject seems irrelevant but easy; e.g., Lazarides et al., 2019; Viljaranta et al., 2016, 2017). However, since these studies applied dispositional measures, it is not yet known whether similar profiles would be found within regard to specific learning situations. The situation-level is important, because it is possible that a student’s motivational profile might change from one learning situation to another.

Moreover, few studies have so far investigated situational aspects of costs [for an exception see Tanaka and Murayama’s (2014) study involving task difficulty], or intra-individual profiles involving values and costs. Although costs are usually expected to be low when other value components are high, this might differ between situations. For example, learning activities could be costly but at the same time enjoyable, or useful, or important to one’s identity (Conley, 2012). Moreover, studies on academic motivation and emotions suggest that positive and negative experience can go hand in hand within learning situations (e.g., Pekrun et al., 2002; Moeller et al., 2015, 2018). For example, Moeller et al. (2015) found that intrinsic motivation was negatively associated with anxiety in some situations and students, but positively associated in others. It can therefore be expected that intrinsic task value and emotional costs occur together in some (*motivating but costly*) situations.

### Situational and Dispositional Motivation

It is not known whether the situational expectancy-value-cost profiles relate to stable motivational dispositions of a student. However, people’s everyday experiences are the driving forces behind development, according to developmental meta-theories, such as dynamic systems theory (e.g., Fogel, 2011; Hollenstein et al., 2013). Vice versa, stable dispositions of personality, attitudes and behavior also constrain and influence the situational experiences people make. This implies that the motivational dispositions that students bring into a learning situation likely affect their motivational experience during learning (e.g., Durik et al., 2015). For instance, a student who generally does not believe in her competence to learn a foreign language might also expect little success in situations when she engages in an actual language learning task (e.g., Trautwein et al., 2009, Study 3). These rationales suggest that repeated experiences of certain situational motivations may influence the development of stable motivational dispositions. This process of crystallizing repeated situational motivation leading to stable dispositions is for example described in Hidi and Renninger’s (2006) model of interest development, where repeated experiences of situational interest are assumed to contribute to the development of individual (dispositional) interest.

### The Present Research

This study is part of the *Momentary Motivation* research project (Dietrich et al., 2017), which measured situational success expectancies and values (including costs) with respect to the learning contents in a university lecture, as well as dispositional competence beliefs, values, and costs that students assign to Educational Psychology as a subject in their undergraduate studies. Earlier analyses of these data (Dietrich et al., 2017) showed considerable intra-individual variability of success expectancies and values.^1^ However, the question to what extent *distinct in-the-moment profiles* of motivation exist, and to what extent these profiles change from one learning situation to another, has not been answered yet.

For the present study, we moreover expected university students’ situational experiences of expectancies, values and costs to be associated with their dispositional expectancies, values and costs in the beginning and in the end of the semester. Students with high (vs. low) dispositional levels of expectancies, values, or costs were expected to show frequent occurrences of motivational situations characterized by similarly high (vs. low) situational expectancies, values, and costs. Moreover, the motivational states experienced during the semester were expected to predict corresponding changes in dispositional expectancies, values and costs from the semester start to the semester end.

We examined the following research questions:

**RQ1: What profiles of situational motivation do university students experience during learning and how do they change?**

We expected to find six different constellations (profiles) of expectancies, values and costs (hypothesis 1). Based on existing findings (Bråten and Olaussen, 2005; Lazarides et al., 2019), we expected three profiles with aligned levels of expectancies, values (i.e., either both high, or both low), and oppositely scored costs. That means we expected that expectancies and values would be either both (1) *high*, or both (2) *low*, or both (3) *moderate*. Corresponding costs were expected to be low when expectancies and values were high, and vice versa. In addition, in line with the findings on dispositional motivation profiles (Viljaranta et al., 2016, 2017; Lazarides et al., 2019), we hypothesized to find two discrepant constellations of (4) *low expectancies and high values* and (5) *high expectancies and low values*. Finally, we expected a constellation of (6) equally high levels of expectancies, values, and costs (“*motivating but costly* situations”; Conley, 2012; Moeller et al., 2015; Salmela-Aro et al., 2016). As part of research question 1 we also examined the transition probabilities from one motivational profile to another, that is, how likely it is to remain in the same profile or to change the profile, from one learning situation to the next.

**RQ2: How are these profiles of situational motivation related to students’ motivational dispositions?**

In line with general dynamic systems theory (Fogel, 2011) and prior research (Trautwein et al., 2009; Durik et al., 2015), we expected that frequent occurrences of high (or low) *situational* expectancies, values, and costs would be associated with similarly high (or low) *dispositional* expectancies, values, or costs in the beginning and in the end of the semester (hypothesis 2a), and with more positive development of these dispositions over the course of the semester (hypothesis 2b). This means, for example, that students with high dispositional expectancies should be particularly prone to experiencing learning situations of either high motivation (profile 1), situations of high expectancies but low values (profile 5), and motivating but costly situations (profile 6), because all these situations are characterized by high situational expectancies. Moreover, students who frequently experience situations of high motivation, or situations of high expectancies but low values or motivating but costly situations during the semester, were expected to show an increase of dispositional expectancies from the beginning to the end of the semester. Table 1 depicts the specific hypotheses comparing frequent occurrences of the different profiles of motivational situations that follow from our general assumption.

**TABLE 1:** Hypothesized relationships between motivational situations and dispositions.

**Level and developmental changes in…**	**…in relation to frequent occurrences of motivational situations**
Dispositional expectancies	Profile 1 > Profile 3 > Profile 2
	Profile 4 < Profile 1/Profile 3
	Profile 5 > Profile 2/Profile 3
	Profile 6 > Profile 2/Profile 3
Dispositional values	Profile 1 > Profile 3 > Profile 2
	Profile 4 > Profile 2/Profile 3
	Profile 5 < Profile 2/Profile 3
	Profile 6 > Profile 2/Profile 3
Dispositional costs^a^	Profile 1 < Profile 3 < Profile 2
	Profile 6 > Profile 1/Profile 3

*Profile 1 = high motivation. Profile 2 = low motivation. Profile 3 = medium motivation. Profile 4 = high value, low expectancy. Profile 5 = low value, high expectancy. Profile 6 = motivating but costly. Descriptions of the expected situational profiles see Research Question 1. ^*a*^No hypotheses were set regarding Profile 4 and 5.*

## Materials and Methods

### Sample and Procedure

The participants were 155 German university students, 51% of which were female (for more information, see Dietrich et al., 2017). The mean age was *M* = 21.77 years (SD = 2.91; range: 19–46 years). The participants studied in a teacher education program with the aim to become subject teachers for secondary schools. We applied a short-term intensive longitudinal design and followed the students over one semester in a weekly lecture with 90-min lessons. The subject of the lecture was “Psychological fundamentals of learning.” It ended with a written exam.

**FIGURE 1:**
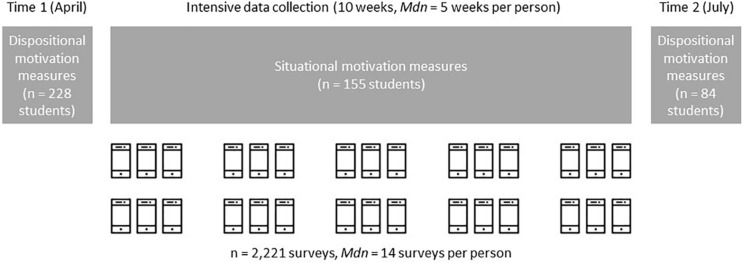
Study design and Ns for the dispositional and situational measurements.

In each of 10 consecutive weeks, students received notifications and questionnaires at fixed schedules, three times during each lesson, consisting of situational motivation items (*intensive data collection*, see Figure 1). The participants chose whether to respond online with their own smartphone or on paper-and-pencil questionnaires (smartphone: 58–71% participants, *M* = 65% across the 10 lessons; paper-and-pencil: 29–42% participants, *M* = 35%).

Participants additionally completed questionnaires assessing dispositional motivation at the semester start (Time 1, April 2014, before the first situational assessment) and semester end (Time 2, July 2014, after the last situational assessment, see Figure 1).

About 400 students attended the first lesson of the lecture. Out of these, 242 students (61%) agreed to participate in the study, and 228 students completed the Time 1 assessment. For each of the lessons in which the situational assessments took place, we determined the proportion of participants in relation to the number of students present. The mean proportion of participants among students present in the lecture was 58% (ranging from 41 to 71%), with no systematic trend over time. However, the absolute number of participants decreased from 151 in the first lesson with situational assessment to 61 in the tenth lesson (*M* = 94.3). Eighty-four students completed the Time 2 assessment.

Overall, 155 students provided valid information on situational measures in at least one lesson. To determine the amount of selectivity in our data, we examined the extent to which the number of lessons a student had attended (number of weeks) was related to the responses in the dispositional motivation measures and some control variables. The number of weeks was largely unrelated to dispositional motivation at Time 1 (−0.13 ≤ *r* ≤ 0.12), unrelated to students’ gender (*r* = 0.04) but positively related to better high school grades (*r* = 0.33). Moreover, the 84 students who provided Time 2 data had reported somewhat higher emotional cost (Cohen’s *d* = 0.20), and lower intrinsic value (*d* = 0.17) and attainment value (*d* = 0.17) at Time 1, compared to those students who did not provide Time 2 data (other differences on dispositional motivation were 0 ≤ *d* ≤ 0.14). Time 2 respondents also had better grades in high school (*d* = 0.27).

The data were subjected to careful data cleaning of each individual row in the data set. This is especially important for the analysis of situational motivation profiles where systematic response patterns could lead to finding biased profiles. During the data cleaning, we removed responses in the following cases: if the response was given more than 15 min after the signal; if a person pretended to be present at the lecture but responded online after the lecture had ended; if a person responded to the three surveys shortly after another; and if a person responded with the same value on all 10 items. This resulted in the omission of 251 surveys. A total of 2,221 valid responses could be used in the analyses, which equals 48% of the possible responses (3 responses per lesson by 10 lessons by 155 participants resulting in 4,650).

### Measures

The situational questionnaire consisted of 10 items, eight of which measured expectancies and task values (Appendix A – available at https://osf.io/qjkmz; see Dietrich et al., 2017, for a factor analysis). The students were instructed to consider the lecture contents of the past couple of minutes and to complete the questionnaire within 10 min. They were then prompted – “To what extent do the following statements apply to you in the present moment?” – and responded on a 4-point Likert scale ranging from 1 = *does not apply* to 4 = *fully applies*. *Situational success expectancies* were measured with two items addressing the expectation of success for the final exam (adapted from Wigfield and Eccles, 2000) and competence experience. Sample item: “I will be good at these contents in the exam” (success expectation). Cronbach’s alpha was 0.65 at the within-level, and 0.77 at the between-level (see below for our data analytic approach using multilevel modeling). *Situational task values* were measured with six items addressing the facets of intrinsic value, utility value for future job, attainment value/personal importance, as well as three subfacets of cost value (effort cost, emotional cost, and opportunity cost). Items were adapted from the task value scale by Gaspard et al. (2015b). Example items were “I like these contents” (intrinsic value), and “Learning these contents exhausts me” (effort cost). We built two composite variables of situational task values: One labeled *Values* comprising the facets intrinsic, attainment, and utility value (Cronbach’s alpha at the within/between level = 0.73/0.89), and one labeled *Costs* comprising its subfacets effort cost, emotional cost, and opportunity cost (Cronbach’s alpha at the within-/between-level = 0.76/0.85).

Dispositional motivation was assessed in the Time 1 and Time 2 questionnaires (Appendix B – available at https://osf.io/qjkmz). *Dispositional success expectancies* were measured with three items on a 4-point Likert scale (1 = *very bad*, 4 = *very good*) asking about the expectation to be successful in the final exam (adapted from Wigfield et al., 1997). Example item: “What do you think, how good will you be at the exam?” Cronbach’s alpha at Time 1/Time 2 was 0.64/0.66. *Dispositional task values* were assessed with items adapted to the present context (Psychology instead of Math) from Gaspard et al. (2015b). Items were rated on a 4-point Likert scale (1 = *does not apply* to 4 = *fully applies*). *Dispositional intrinsic value* was assessed with four items. Example item: “I enjoy dealing with psychological topics.” Cronbach’s alpha at Time 1/Time 2 was 0.84/0.87. *Attainment value* was assessed with eight items. Example item: “It is important to me to know a lot of Psychology.” Cronbach’s alpha was 0.86/0.88. *Utility value* was measured with the utility for future job subscale (4 items). Example item: “Good knowledge in Psychology will be useful in my future occupation.” Cronbach’s alpha was 0.77/0.81. *Effort cost* was assessed with three items. Example item: “Dealing with Psychology drains a lot of my energy.” Cronbach’s alpha was 0.76/0.82. *Emotional cost* was assessed with four items. Example item: “Psychology is a real burden to me.” Cronbach’s alpha was 0.69/0.79. Finally, *opportunity cost* was assessed with three items. Example item: “I have to give up other activities that I like to be successful at Psychology.” Cronbach’s alpha was 0.72/0.87.

### Analytic Strategy

#### Multilevel Latent Profile Analysis

Given the hierarchical structure of the data, we conducted multilevel analyses with learning situations (level 1, *n* = 2,221) nested in students (level 2, *n* = 155). In order to identify clusters of situations with similar profiles of expectancies, task values, and costs (RQ1), we conducted a MLPA (Henry and Muthén, 2010; Appendix C – available at https://osf.io/qjkmz). The goal of this MLPA is to identify latent profiles that best describe the patterns of the indicator variables within learning situations (within, situation level) while simultaneously taking into account the nesting of data within students (between-level, dispositions). For example, in some individuals the probability of being in a certain situational motivation profile might be higher than in other individuals. In an MLPA model, this is represented in estimating the between-level variance of the latent class means (parametric approach). In the case of three or more latent classes, the random means on the between-level are correlated with one another, but because modeling such correlations is computationally very heavy, it is recommended to use a common factor to model the associations of the random means (Henry and Muthén, 2010). Moreover, to keep the computation feasible, in the MLPA models the variances were held equal across latent classes and expectancies, task values, and costs were uncorrelated within class.^2^

A model with the adequate number of latent profiles was determined based on information criteria [BIC, sample size adjusted BIC (SABIC), and AIC] and the interpretability of the model (Henry and Muthén, 2010). We also inspected the entropy, which indicates the precision with which the situations are classified into the profiles.

#### Probability Sampling

After choosing a final model, we applied a probability sampling procedure for the further analyses (Sahdra et al., 2017; Appendix D – available at https://osf.io/qjkmz). This procedure is an alternative to saving the most likely latent profile membership and takes into account the uncertainty that is associated with classifying learning situations into the latent profiles. The probability sampling was based on the profile probabilities (i.e., the probability that a learning situation belongs to each of the latent profiles). From these distributions, we sampled 25 data sets containing profile membership information that were analyzed separately in the following analyses. Finally, the results were aggregated appropriately across the parameter estimates obtained in each of the 25 model runs (Rubin, 1987).

#### Multilevel Regression

To examine the associations between the profiles of situational motivation and students’ dispositional motivation (RQ2), we again used multilevel modeling to examine the motivational profiles (latent profile membership) on both the within-level and the between-level, and dispositional motivation on the between-level (Appendix E – available at https://osf.io/qjkmz). On the within-level, latent profile membership indicates that a given learning situation belongs to a certain situational motivation profile. Profile membership on the between-level indicates an individual’s propensity to experience each of the motivational profiles.

To examine hypothesis 2a, we regressed dispositional motivation at the beginning (Time 1) and end of the semester (Time 2) on profile membership (*profile differences model*). The resulting regression coefficients represent comparable estimates for the association between situational profile memberships and Time 1/Time 2 motivation. To examine hypothesis 2b, we modeled the change score (difference) from Time 1 to Time 2. The change score was then regressed on latent profile membership (*differential change model*). We estimated separate models for dispositional expectancies, dispositional values, and dispositional costs (6 models in total), all of which were saturated.

In all analyses, we dealt with missing data using full-information maximum likelihood estimation, which uses all available data without imputing missing values (Schafer and Graham, 2002).

## Results

### Measurement Invariance for Dispositional Motivation

We tested for measurement invariance of all dispositional constructs across time using confirmatory factor analysis (CFA). We determined measurement invariance following Cheung and Rensvold (2002) who suggested that if the decrease in CFI is not more than 0.01, and the RMSEA increases by less than 0.015 for the more parsimonious model, then invariance can be assumed. Table 2 shows the fit indices for the most invariant model for each construct. Full scalar invariance held for all constructs except emotional cost, where we used a partial invariance model. For each construct, factor scores from the most invariant model were saved and used in the primary analyses. For the emotional cost subscale, the CFA revealed that the loading and intercept of one item were not invariant over time (“When I deal with Psychology, I get annoyed”). Thus, the factor scores for this scale were based on a model with partial invariance.

**TABLE 2:** Descriptive statistics and model fit information for tests of measurement invariance of the dispositional motivation measures.

**Construct**	***M* (SD)**		***r*_Time 1,Time 2_**	**χ^2^**	***df***	**RMSEA**	**CFI**	**TLI**	**Note**
								
	**Time 1**	**Time 2**							
Expectancy for success	2.82 (0.25)	2.80 (0.32)	0.97	16.77	12	0.04	0.97	0.97	Full scalar invariance
Intrinsic value	2.95 (0.50)	3.03 (0.52)	0.60	26.29	25	0.01	1.00	1.00	Full scalar invariance
Attainment value	3.00 (0.40)	3.03 (0.45)	0.78	147.44	117	0.03	0.96	0.96	Full scalar invariance
Utility value	3.08 (0.42)	3.14 (0.42)	0.57	40.44	25	0.05	0.94	0.93	Full scalar invariance
Effort cost	1.99 (0.52)	1.91 (0.54)	0.68	18.75	12	0.05	0.97	0.96	Full scalar invariance
Emotional cost	1.49 (0.39)	1.32 (0.47)	0.58	40.75	23	0.06	0.93	0.92	Partial metric and scalar invariance
Opportunity cost	1.98 (0.46)	1.99 (0.64)	0.66	24.10	12	0.06	0.95	0.94	Full scalar invariance

**M*, SD, *r* based on factor scores.*

### Descriptive Statistics for Situational Motivation

Before carrying out our substantial analyses, we computed intra-class correlations (ICCs) to determine the amount of variability that was due to different situations (within-level) and students (between-level). Latent variable ICCs were 0.31 for expectancies, 0.36 for values, and 0.55 for costs. This substantial amount of variance that was due to differences between students justified the multilevel approach used for further analyses. Situational expectancies and values were generally higher rated than costs (expectancies: *M* = 3.11; values: *M* = 2.97; costs: *M* = 1.75). This implies that students on average affirmed experiencing success expectancies and values, while they on average denied the items asking about costs. Situational expectancies and values were positively correlated with each other (*r* = 0.55) and negatively correlated with costs (−0.33 ≤ *r* ≤ −0.31).

### Latent Profile Analyses (RQ1)

**FIGURE 2:**
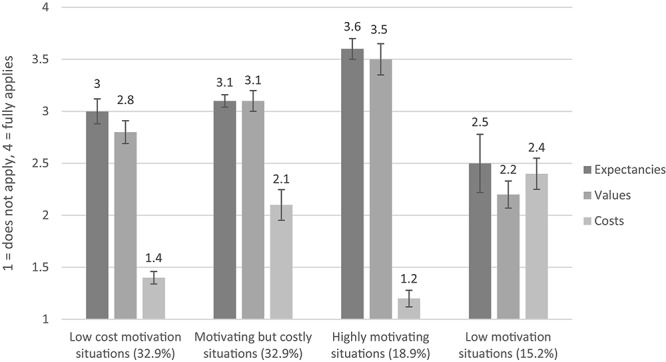
Profiles of students’ motivational experience in learning situations. Depicted are means and confidence intervals.

Table 3 shows the model fit indices for latent profile models with up to six profiles. Although the information criteria suggested to add more latent profiles, the best likelihood did not replicate in the 5- and 6-profile models.^3^ The results of the MLPA revealed that in most situations, expectancies and values were aligned, meaning both scores were similarly high, or similarly moderate, or both low in most situations, while cost scores were low when values and expectancies were high, and vice versa. Based on the model fit indices and interpretability, we chose the 4-profile model with the following profiles of students’ situational motivation (see Figure 2). As expected in hypothesis 1, we found three profiles with symmetric expectancies, values, and costs: A profile of medium expectancies and values, and low costs (“*low cost motivation situations*,” 32.9% of situations), a profile of high expectancies and values, and low costs (“*highly motivating situations*,” 18.9%), and a profile of low expectancies and values, and above average costs (“*low motivation situations*,” 15.2%). Further as expected, we found a profile of medium expectancies and values, and above average costs that resembled “*motivating but costly situations*” (32.9%). In contrast to hypothesis 1, we did not find the expected discrepant constellations of high expectancies and low values, and vice versa.

**TABLE 3:** Model fit information for multilevel latent profile analysis.

**No. profile**	**LL**	**BIC**	**SABIC**	**AIC**	**Entropy**
1	–5549.14	11144.51	11125.45	11110.27	–
2	–5194.60	10466.25	10434.48	10409.19	0.67
3	–4540.88	9205.05	9145.22	9113.76	0.83
4	–4333.01	8827.84	8761.12	8708.02	0.82
5	–3837.063	7874.47	7791.87	7726.13	0.99
6	–3745.23	7729.32	7630.83	7552.45	0.95

*LL = Loglikelihood. In the models with five and six profiles, the best LL was not replicated.*

To examine change and stability in situational profile membership, we computed transition probabilities from one motivational profile to the next within one lecture session. Table 4 shows these transition probabilities (based on probability sampling, see section “Analytic Strategy”). As the table shows, a student’s motivational profile was relatively stable, reflected in the probabilities of 0.61–0.83 for staying in the same motivational profile. Changes mostly occurred between the *high motivation* and the *low cost motivation* profiles (probabilities of 0.26 and 0.16), and between the *low motivation* and the *motivating but costly* profiles (probabilities of 0.21 and 0.11), being in both directions.

**TABLE 4:** Transition probabilities across profiles within one lecture session.

		**Class membership time *t* + 1**			
		
		**Low cost motivation situations**	**Motivating but costly situations**	**Highly motivating situations**	**Low motivation situations**
Class membership time *t*	Low cost motivation situations	0.79	0.02	0.16	0.03
	Motivating but costly situations	0.02	0.83	0.03	0.11
	Highly motivating situations	0.26	0.07	0.61	0.06
	Low motivation situations	0.05	0.21	0.07	0.67

### Associations Between Situational and Dispositional Motivation (RQ2)

#### Profile Differences at the Beginning and End of the Semester

Next, we tested whether the experiences of motivational states (latent profiles) were associated with dispositional expectancies, values, and costs at the beginning and end of the semester (*profile differences models*). The results are depicted in Table 5.

**TABLE 5:** Associations between profiles of situational motivation and dispositional constructs.

**Dispositional construct**	**Situational profiles**	**Profile differences model**				**Differential change model**	
			
		**Time 1**		**Time 2**		**Change Time 1–Time 2^1^**	
				
		**β**	***p***	**β**	***p***	**β**	***p***
Expectancy for success	Low vs. low cost	–0.046	0.676	–0.047	0.728	0.008	0.875
	Motivating but costly vs. low cost	0.083	0.258	0.071	0.441	–0.006	0.855
	High vs. low cost	**0.281**	**0.047**	0.286	0.125	0.010	0.871
	Motivating but costly vs. low	0.129	0.277	0.118	0.419	–0.013	0.793
	High vs. low	**0.328**	**0.047**	0.333	0.118	0.002	0.971
	High vs. motivating but costly	**0.199**	**0.044**	0.215	0.112	0.016	0.746
Intrinsic value	Low vs. low cost	–0.214	0.257	**−0.600**	**0.016**	–0.386	0.067
	Motivating but costly vs. low cost	0.116	0.335	0.238	0.106	0.123	0.363
	High vs. low cost	**0.504**	**0.026**	**1.043**	**> 0.001**	0.540	0.055
	Motivating but costly vs. low	0.330	0.114	**0.838**	**0.001**	**0.508**	**0.030**
	High vs. low	**0.718**	**0.009**	**1.643**	**> 0.001**	**0.925**	**0.005**
	High vs. motivating but costly	**0.388**	**0.033**	**0.805**	**> 0.001**	0.417	0.051
Attainment value	Low vs. low cost	**−0.305**	**0.018**	**−0.524**	**0.005**	–0.219	0.120
	Motivating but costly vs. low cost	**0.213**	**0.037**	**0.410**	**> 0.001**	**0.197**	**0.016**
	High vs. low cost	**0.591**	**> 0.001**	**10.045**	**> 0.001**	**0.453**	**0.018**
	Motivating but costly vs. low	**0.518**	**> 0.001**	**0.934**	**> 0.001**	**0.416**	**0.007**
	High vs. low	**0.896**	**> 0.001**	**1.569**	**> 0.001**	**0.673**	**0.003**
	High vs. motivating but costly	**0.378**	**0.004**	**0.635**	**> 0.001**	0.256	0.085
Utility value	Low vs. low cost	–0.266	0.088	−**0.499**	**0.006**	–0.233	0.149
	Motivating but costly vs. low cost	**0.255**	**0.018**	0.167	0.179	–0.088	0.460
	High vs. low cost	**0.477**	**0.022**	**0.670**	**0.004**	0.193	0.391
	Motivating but costly vs. low	**0.520**	**0.002**	**0.666**	**0.002**	0.145	0.465
	High vs. low	**0.743**	**0.002**	**10.169**	**> 0.001**	0.425	0.125
	High vs. motivating but costly	0.223	0.166	**0.503**	**0.011**	0.280	0.146
Effort cost	Low vs. low cost	0.198	0.323	0.425	0.104	0.227	0.315
	Motivating but costly vs. low cost	0.228	0.081	0.250	0.084	0.022	0.865
	High vs. low cost	**−0.572**	**0.025**	–0.538	0.064	0.034	0.901
	Motivating but costly vs. low	0.030	0.883	–0.175	0.563	–0.205	0.411
	High vs. low	**−0.770**	**0.005**	**−0.963**	**0.010**	–0.193	0.557
	High vs. Motivating but costly	**−0.800**	**> 0.001**	**−0.788**	**0.001**	0.012	0.955
Emotional cost	Low vs. low cost	0.277	0.092	0.385	0.077	0.108	0.493
	Motivating but costly vs. low cost	0.148	0.099	0.072	0.627	–0.076	0.559
	High vs. low cost	**−0.416**	**0.005**	–0.441	0.135	–0.025	0.914
	Motivating but costly vs. low	–0.129	0.495	–0.313	0.203	–0.184	0.337
	High vs. low	**−0.693**	**0.002**	**−0.826**	**0.009**	–0.133	0.551
	High vs. motivating but costly	**−0.565**	**> 0.001**	**−0.514**	**0.018**	0.051	0.787
Opportunity cost	Low vs. low cost	0.320	0.085	0.529	0.059	0.210	0.325
	Motivating but costly vs. low cost	**0.282**	**0.013**	**0.388**	**0.042**	0.106	0.490
	High vs. low cost	**−0.456**	**0.027**	**−0.749**	**0.025**	–0.293	0.291
	Motivating but costly vs. low	–0.038	0.853	–0.141	0.660	–0.104	0.689
	High vs. low	**−0.775**	**0.002**	**−1.278**	**0.001**	–0.503	0.110
	High vs. motivating but costly	**−0.738**	**> 0.001**	**−1.137**	**> 0.001**	–0.399	0.098

*Unstandardized between-level regression coefficients for pairwise comparisons between profiles (the second category is the reference category). High = *highly motivating situations*. Motivating but costly = *motivating but costly situations*. Low = *low motivation situations*. Low cost = *low cost motivation situations*. ^1^The change score is the difference between Time 1 and Time 2. Statistically significant effects printed in bold.*

As predicted in hypothesis 2a, students who often experienced *highly motivating situations* during the lecture reported higher success expectations in the beginning of the semester (Time 1), compared to frequent experiences of all other situational profiles.

Concerning task values, students who often experienced *highly motivating situations* (high expectancies and values, and low costs) during the lecture tended to hold higher intrinsic, attainment, and utility value both at Time 1 and Time 2, compared to frequent experiences of all other situational profiles, which is again in line with hypothesis 2a. An exception was utility value at Time 1, which did not differ between students who often experienced *highly motivating* versus *motivating but costly situations* (medium expectancies and values, and above average costs). In contrast, frequent experiences of *low motivation* (low expectancies and values, and above average costs) were associated with lower attainment and utility value at both Time 1 and Time 2, and with lower intrinsic value at Time 2, compared to all other situational profiles. Finally, students who often experienced *motivating but costly situations* held higher attainment and utility values at Time 1, and reported higher attainment value at Time 2 than students with frequent *low cost motivation situations*.

Also as predicted, we found that students with frequent experiences of *high motivation* during the lecture had lower effort, emotional, and opportunity costs than other students at both Time 1 and Time 2. These students did, however, not differ significantly at Time 2 from students with frequent *low cost motivation situations* on effort cost and emotional cost. Moreover, students who often experienced *motivating but costly situations* reported higher opportunity cost at Time 1 and Time 2, compared to students with frequent *low cost motivation situations*.

#### Differential Change

Next, we examined whether experiences of situational profiles predicted changes in dispositional motivation over the course of the semester (*differential change models*, see Table 5). We found differential change in task values, but not in regard to expectancies and costs. The results showed that frequently experiencing *highly motivating situations* was associated with more positive change in intrinsic and attainment value, compared to frequent experiences of other motivational situations. Moreover, students who often experienced *motivating but costly situations* tended to show more positive change in attainment value compared to students with frequent experiences of *low cost motivation* or *low motivation situations*. Also, students with frequent experiences of *motivating but costly situations* increased their intrinsic value relative to students with frequently *low motivation*. These findings concerning task values were in line with hypothesis 2b.

## Discussion

When teachers aim to provide a learning environment which optimally fosters engagement in learning they need to take into account that students not only bring different (motivational) dispositions, but also vary over time in their motivational state. This study combined intensive data with a short-term longitudinal design to examine university students’ motivational beliefs on the state and trait level. We identified four different profiles of expectancies, values, and costs within specific learning situations during a university course and examined situational change and stability. A student’s frequency of experiencing certain situational profiles was associated with that student’s dispositional motivation towards the topic of the lecture.

### Profiles of Motivational Experiences

In accord with our expectations, our findings suggest that students experience most learning situations in terms of similar expectancies and values, and opposite levels of costs – regardless of their overall level of motivation (high, medium, or low) in that situation. In *high motivation situations*, high expectancies and values occurred together with low costs; in *low motivation situations*, low expectancies and values occurred with above average costs; and in *low cost motivation situations*, medium expectancies and values occurred with low costs. Together, these symmetric profiles accounted for 67.0% of the learning situations in the lecture. Our situation-level results corroborate earlier studies on dispositional motivation reporting mainly such aligned profiles of expectancies and values (e.g., Bråten and Olaussen, 2005; Dietrich and Lazarides, 2019; Lazarides et al., 2019) and correspond with correlational research reporting a positive linear relationship between expectancies and values (e.g., Denissen et al., 2007, Kosovich et al., 2017).

Moreover, there was one profile in which medium values and expectancies occurred together with above average levels of costs (*motivating but costly situations*). Although this profile was relatively frequent (33% of all situations), it would have been overlooked if only correlations had been examined (due to the negative correlations of costs with expectancies and other values; −0.33 ≤ *r* ≤−0.31). This finding underscores the particular merit of analyzing profiles. A co-occurrence of heightened values with heightened costs has been described before for secondary students’ dispositional math motivation (Conley, 2012). The finding also aligns with existing studies describing co-occurring positive and negative aspects of academic emotions (Pekrun et al., 2002; Moeller et al., 2015, 2018) and other motivational constructs such as school engagement (Tuominen-Soini and Salmela-Aro, 2014; Salmela-Aro et al., 2016).

The in-the-moment profiles of motivational beliefs tended to be relatively stable from one learning situation to the next within one lecture session. However, for students in the *high motivation* profile there was a chance of 0.26 to decrease their expectancies and values 30 min later, and to move to the *low cost motivation* profile. But also for students in the *low motivation* profile, the chances were 0.21 to increase expectancies and values, and to move to the *motivating, but costly* profile. Overall, our data suggest that if a student changed her motivational profile, that change more likely occurred to a profile with similar levels of costs but higher or lower expectancies and values. This might indicate that situational expectancies and values could be more malleable to change than situational costs. A fruitful avenue for future research would be to examine the contextual characteristics of the learning situations which prompt shifts in motivational profiles. Information from such studies could be useful in the context of adaptive teaching (Corno, 2008) where teachers adapt their instruction based on the (motivational) needs of certain groups of students. Our study is a first step into this direction showing the motivational heterogeneity in students’ situational experiences. Still it remains an open question for future studies to clarify the extent to which profile change and stability can be attributed to individual and context characteristics.

Contrary to our expectation, we did not find situational profiles with discrepant expectancies and values (e.g., situations of low expectancies and high values, meaning relevant but difficult situations, or low expectancies and high values, meaning irrelevant but easy situations). This is in contrast to previous studies that found that some students reported high levels of self-concept but low levels of interest for a given subject, or vice versa (Viljaranta et al., 2016, 2017; Lazarides et al., 2019). It could be that this difference in finding is due to differences in measures (situational, asking about the current learning situation versus dispositional, asking about the entire subject), or due to different age groups studied, since the correlations between expectancies, values, and costs are typically lower in children than in older students (Wigfield et al., 2009), leaving more possibilities that diverging profiles exist (see, e.g., Viljaranta et al., 2016). Other differences between our study and earlier ones are that we studied motivation in a university Psychology course, while Lazarides et al. (2019) and Viljaranta et al. (2016) studied Math or Reading. Our findings could also be influenced by the fact that the university students in our sample were not obliged to attend the lecture, whereas school students do have to attend classes. It is possible that the students with high expectancies but low values, who think that learning Psychology is easy but unimportant, decide to not attend lessons and only engage in self-study to prepare for the exam.

### Associations Between Situational and Dispositional Motivation

Finally, situational profiles were associated with students’ dispositional motivation at the beginning and at the end of the semester, as well as students’ change in dispositional motivation from the beginning to the end of the semester.

Students’ general values and success expectancy about the study subject predicted their situational experiences during lecture sessions. For example, students with higher dispositional success expectations, higher dispositional values, and lower dispositional costs were more likely to experience states of high motivation during the lecture. Moreover, our findings partly support our hypothesis that a student’s situational motivational states relate to changes in dispositional motivation. Students who frequently experienced low motivation in the lecture showed more negative development in their intrinsic and attainment value towards the subject Psychology, compared to other students. However, this only applied to task values, but not to expectancies or costs. It is thus possible that students’ dispositional value beliefs about a subject are more malleable in the university lecture context, i.e., influenced by students’ everyday learning experiences, than dispositional expectancy or cost beliefs. In teacher-centered university lectures like the one studied here, which are characterized by direct instruction and rather little student activity, the teacher can increase value by stressing the relevance/utility of the content (e.g., Gaspard et al., 2015a) and using intellectual stimulation (e.g., Bolkan and Griffin, 2018). By contrast, typical strategies of changing expectancy beliefs through feedback and attribution (e.g., Perry et al., 2014) might be less applicable in this context.

Students with frequent *motivating but costly situations* strengthened their dispositional task values (attainment, partly intrinsic) more strongly than most other students, except those with frequent high motivation. At the same time, these students with many motivating but costly experiences also reported higher dispositional costs of learning Psychology, compared to many other students, both in the beginning and at the end of the semester.

Not much is known so far about the long-term development of students with co-occurring positive and negative motivation. Longitudinal findings by Tuominen-Soini and Salmela-Aro (2014) suggested that students who experience intrinsic motivation/engagement together with symptoms of exhaustion in adolescence are more likely to develop stronger symptoms of burnout and depression in the long run and to downgrade their educational aspirations as young adults. However, the students in the current study who frequently had motivating but costly experiences might just have a realistic view on education involving a “no pain, no gain” attitude: It is of value (even increases in value) to engage in learning, but it typically has some costs.

Overall, the results of this study support the notions of dynamic systems theory (e.g., Fogel, 2011; Hollenstein et al., 2013) and are in line with some earlier research on the association between situational and dispositional motivation (Trautwein et al., 2009; Durik et al., 2015). Indeed the motivational dispositions that students bring into a learning situation may affect their motivational experience during learning, and in the case of task values, such situational motivation experience may contribute to the development of inter-individual differences in more stable motivational beliefs. However, our findings indicate that situational experience contributed mainly to pre-existing stability in dispositional motivation, and only in the case of values in increased inter-individual differences.

### Directions for Future Research

To our knowledge, this study is among the first to examine profiles of situational expectancies, values and costs in specific learning situations, and to relate such situational profiles to students’ development in dispositional motivation. However, some limitations of this study give suggestions for future research.

First, we investigated expectancies, values, and costs in only one context and population, namely adult teacher students experiencing an introductory lecture about Educational Psychology. It is thus unclear whether these findings can be generalized to students learning in schools or other, more active learning forms, such as group work. Replications in more diverse and representative student samples could examine whether different learning contexts elicit similar profiles as described here, or if not, which exact person and context characteristics determine the situation-level profiles of expectancies, values, and costs. In particular, we did not find difficult but valuable situations but expect that they occur in more active and challenging learning situations. It would therefore be interesting to compare our findings to students’ experiences in more difficult learning situations, such as during a preparation for a Math competition.

Second, while this study was based on a relatively large number of situational observations, the sample size on the level of students was rather small (*N* = 155) and, as is typical in university lectures, declined over the semester, which limited the power in the analyses on motivational dispositions and may have led to selectivity bias. Also, the small sample precluded us from conducting profile analyses on the level of students. It would thus be interesting to compare situational (within-person) and dispositional (between-person) profiles in a larger sample in the future (Voelkle et al., 2014).

Third, in our multilevel analyses, the learning situations were treated as interchangeable measurements. This ignored any developmental dynamics that might have happened during the semester. One such dynamic might be a higher motivational variability in the beginning of a course when the subject and the teacher are new, while later on after making repeated experiences, certain motivational states might occur more easily or more rarely in certain students.

Finally, while the design of this study enabled us to investigate within-lesson changes of motivational profiles, the here reported findings are bound to the small time period between the measurements (30 min). Because some other studies suggest that more variance in state motivation and emotions can be attributed to particular learning situations than for example to different days of the week (e.g., Martin et al., 2015; Ketonen et al., 2018), more and varying designs with within-day sampling intervals seem promising to further our understanding of change and stability in motivation.

Taken together, studying expectancies, values, and costs in the moments in which they occur helps to understand how students’ in-class experiences contribute to their long-term motivational development. Moreover, intra-individual motivational profiles revealed that in some learning situations, positive and negative aspects of motivation co-occurred (in *motivating but costly situations*), which is easily overlooked in the typically applied correlational analyses. How such mixed motivation relates to educational outcomes in the short- or long-term (see Guo et al., 2016), is a further avenue for future research on motivation in actual learning situations.

## Ethics Statement

This study was carried out in accordance with the recommendations of the APA Code of Ethics with written informed consent from all subjects. All subjects gave written informed consent in accordance with the Declaration of Helsinki. At the time of conducting the study (2014) the approval of an ethics committee was not required in Germany.

## Author Contributions

JD, JM, and JV contributed to the conception and design of the project. JD collected the data, with help of JM and BK. JD conducted the statistical analyses, supported by JG. JD and JM wrote the first draft of the manuscript. All authors contributed to the manuscript revision, read and approved the final version of the manuscript.

## Conflict of Interest Statement

The authors declare that the research was conducted in the absence of any commercial or financial relationships that could be construed as a potential conflict of interest.

## Footnotes

1 Dietrich et al. (2017) examined the amount of variance in motivational beliefs that was due to intra- versus inter-individual differences. They also studied intra- and inter-individual associations with effort.

2 As a sensitivity analysis, we ran a series of additional LPA models. These models examined whether similar results would be found when instead of composite scores of expectancies, task values, and costs the single items would be used. In the multilevel context, using single items led to issues with non-convergence from three latent classes upwards. Therefore, we also tested these models as single-level models ignoring the nesting of the data. The results from these models suggested similar latent classes as those described in the “Results” section.

3 The issue of non-convergence could not be solved by increasing the number of iterations to 10,000.
